# Effects of a Multi-Ingredient Preworkout Supplement Versus Caffeine on Energy Expenditure and Feelings of Fatigue during Low-Intensity Treadmill Exercise in College-Aged Males

**DOI:** 10.3390/sports8100132

**Published:** 2020-09-25

**Authors:** Daniel J. Lutsch, Clayton L. Camic, Andrew R. Jagim, Riley R. Stefan, Brandon J. Cox, Rachel N. Tauber, Shaine E. Henert

**Affiliations:** 1 Department of Kinesiology and Physical Education, Northern Illinois University, DeKalb, IL 60115, USA; Z1734695@students.niu.edu (D.J.L.); rstefan1@niu.edu (R.R.S.); Z1836633@students.niu.edu (B.J.C.); rtauber1@niu.edu (R.N.T.); shenert@niu.edu (S.E.H.); 2 Sports Medicine, Mayo Clinic Health System, Onalaska, WI 54650, USA; jagim.andrew@mayo.edu

**Keywords:** supplementation, ergogenic aid, metabolic rate, energy, substrate utilization

## Abstract

The primary purpose of this study was to examine the acute effects of a multi-ingredient (i.e., caffeine, green tea extract, Yohimbe extract, capsicum annum, coleus extract, L-carnitine, beta-alanine, tyrosine) preworkout supplement versus a dose of caffeine (6 mg·kg^−1^) on energy expenditure during low-intensity exercise. The effects of these treatments on substrate utilization, gas exchange, and psychological factors were also investigated. Twelve males (mean ± SD: age = 22.8 ± 2.4 years) completed three bouts of 60 min of treadmill exercise on separate days after consuming a preworkout supplement, 6 mg·kg^−1^ of caffeine, or placebo in a randomized fashion. The preworkout and caffeine supplements resulted in significantly greater energy expenditure (*p* < 0.001, *p* = 0.006, respectively), $\dot{V}O$_2_ (*p* < 0.001, *p* = 0.007, respectively), $\dot{V}$CO_2_ (*p* = 0.006, *p* = 0.049, respectively), and $\dot{V}$_E_ (*p* < 0.001, *p* = 0.007, respectively) compared to placebo (collapsed across condition). There were no differences among conditions, however, for rates of fat or carbohydrate oxidation or respiratory exchange ratio. In addition, the preworkout supplement increased feelings of alertness (*p* = 0.015) and focus (*p* = 0.005) 30-min postingestion and decreased feelings of fatigue (*p* = 0.014) during exercise compared to placebo. Thus, the preworkout supplement increased energy expenditure and measures of gas exchange to the same extent as 6 mg·kg^−1^ of caffeine with concomitant increased feelings of alertness and focus and decreased feelings of fatigue.

## 1. Introduction

Multi-ingredient preworkout supplementation has become a popular strategy for individuals attempting to improve feelings of energy and focus; muscular strength; endurance; muscle mass; blood flow; and fat loss during resistance and aerobic training, group exercise classes, as well as recreational activities [1]. Among the major concerns regarding preworkout supplements, however, is the common manufacturing practice of using numerous, relatively obscure ingredients often combined into proprietary blends at unknown quantities [2]. A recent study [2] examined the common ingredient profiles of the top 100 best-selling preworkout supplements and reported that these products contain 18.4 ± 9.7 (mean ± SD) ingredients with the majority including beta-alanine (87%), caffeine (86%), citrulline (71%), tyrosine (63%), and taurine (51%). Their findings [2] indicated that 58% of these preworkout supplements have at least one proprietary blend with 64 ± 24% of all ingredients listed at undisclosed quantities. Moreover, of the five ingredients (i.e., beta-alanine, caffeine, citrulline, creatine, and arginine) with well-established recommended doses, caffeine was the only ingredient provided at an average quantity at or above its suggested ergogenic threshold [2].

Caffeine is a widely-used, mild central nervous system stimulant that blocks adenosine receptors [3] and has been shown to improve a number of metabolic, psychological, and neuromuscular factors during exercise with the administration of 3–6 mg·kg^−1^ of body mass [4,5]. For example, caffeine has been demonstrated to increase free fatty acid mobilization [6] and energy expenditure [7,8]. In addition, it has been reported that caffeine ingestion enhances psychological functions including increased feelings of focus and alertness [9,10], reduced feelings of exertion [11,12], and improved mental efforts and cognition [13]. These effects translate into diverse performance benefits during low-to-high intensity exercise such as increased time to exhaustion [14], neuromuscular function [15], skeletal muscle contraction capabilities [15], repetitions to failure of the chest press [16], and peak power on the Wingate anaerobic test [16]. Based on these findings [3,4,5,6,7,8,9,10,11,12,13,14,15,16], caffeine alone may be favorable to consumers, provided that it is a well-established ergogenic aid with specific dosing recommendations and free of the proprietary blends typically associated with preworkout supplements.

The growing prevalence of preworkout supplementation is based on the purported synergistic effect of multiple ingredients [17]. Previous studies have shown that acute ingestion of a preworkout supplement has improved total resistance training volume [18,19,20,21], repetitions to failure for squat [22] and bench press [19,21,23], and upper body peak and mean power [20]. Currently, the majority of research on preworkout supplementation has been associated with muscular endurance and anaerobic performance measures, whereas there are limited data concerning aerobic activity. The findings of Walsh and colleagues [17], however, indicated that ingestion of preworkout increased time to exhaustion in treadmill running at 70% $\dot{V}O$_2max_ and concurrently improved feelings of energy and focus. It is possible that these findings [17] were entirely due to the inclusion of caffeine in the multi-ingredient preworkout supplement. Thus, despite the multitude of ingredients in preworkout supplements, and inclusion of specialized proprietary blends of various compounds, it is possible that any ergogenic effects of these products are solely due to caffeine, if provided at sufficient levels. Moreover, several of the included ingredients common to preworkout supplements often require multiple days to weeks to confer any ergogenic benefit, again suggesting that any acute performance benefit may be attributable to caffeine. Therefore, the primary purpose of this study was to examine the acute effects of a multi-ingredient preworkout supplement versus a dose of caffeine (6 mg·kg^−1^) on energy expenditure during low-intensity exercise. In addition, we investigated the effects of these substances on substrate utilization (i.e., fat and carbohydrate utilization), gas exchange, and psychological factors (subjective feelings of energy, fatigue, focus, and alertness).

## 2. Materials and Methods

### 2.1. Study Design

**Figure 1:**
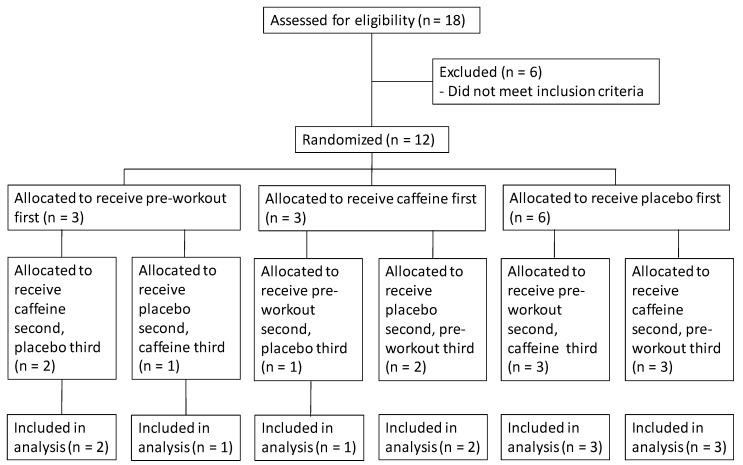
Consolidated Standards of Reporting Trials (CONSORT) flow diagram.

This study utilized a randomized, double-blind, placebo-controlled, within-subjects crossover design (Figure 1). Subjects were required to visit the laboratory on four occasions separated by 48–72 h. During the first laboratory visit, each subject performed a 5-min walk on a motorized treadmill at speeds of 4.8–6.4 km·h^−1^ to familiarize the subjects with the testing procedures. For the second visit, each subject came to the laboratory (06:00 a.m.–08:00 a.m.) following an overnight fast of at least 8 h and was randomly assigned to ingest: (1) one serving of the preworkout supplement, (2) 6 mg of caffeine per kg of body mass, or (3) one serving of the placebo 30 min before the exercise protocol. Immediately before ingestion, 30 min postingestion, and at the 30-min time point of exercise, subjects completed a questionnaire to determine feelings of fatigue, energy, focus, and alertness. After completion of the questionnaire, subjects performed a 60-min constant-velocity treadmill walk at a self-selected intensity of 4.8–6.4 km·h^−1^. Subjects returned for their third and fourth visits to ingest the remaining substances (preworkout, caffeine, or placebo) and complete identical testing procedures (including time of day and treadmill velocity) as the second visit. All participants were required to complete two-day dietary history logs before each visit to the laboratory using MyFitnessPal (Baltimore, MD, USA).

### 2.2. Subjects

Twelve male subjects (mean ± SD: age = 22.8 ± 2.4 years (range = 19–27 years; 95% CI = 21.5–24.2 years); body mass = 82.7 ± 8.6 kg (range = 68.4–103.3 kg; 95% CI = 77.8–87.5 kg); height = 176.3 ± 8.8 cm (range = 162.5–193.3 cm; 95% CI = 171.3–181.3 cm)) defined as recreationally-trained (resistance training = 4.4 ± 1.8 h·wk^−1^ (range = 0.0–7.0 h·wk^−1^; 95% CI = 3.4–5.4 h·wk^−1^); aerobic training = 1.4 ± 1.4 h·wk^−1^ (range 0.0–4.8 h·wk^−1^; 95% CI = 0.6–2.2 h·wk^−1^)) volunteered to participate in this investigation. The subjects were active in recreational basketball (n = 4), soccer (n = 1), football (n = 1), baseball (n = 1), softball (n = 1), 5 K running races (n = 1), and spartan races (n = 1). The subjects were eligible to participate if they did not report: (i) cardiovascular disease, metabolic, renal, hepatic, or musculoskeletal disorders; (ii) use of any medications; (iii) use of nutritional supplements; (iv) habitual use of caffeine (≥1 caffeinated beverage per day); (v) participation in another clinical trial or investigation of another investigational product within 30 days prior to screening/enrollment; (vi) use of nicotine products. Subjects were instructed to maintain their normal dietary habits and exercise regimens for the duration of their participation. The subjects were instructed to refrain from eating or drinking anything other than water 8 h before laboratory visits 2–4. Additionally, the subjects were asked to completely abstain from caffeine use for at least two weeks before starting the investigation. This study was approved by the Institutional Review Board (Protocol # HS19-0163), registered on clinicaltrials.gov (NCT04539054), and all the participants completed a health history questionnaire and signed an informed consent document before all testing.

#### 2.2.1. Visit 1: Familiarization

The initial visit to the laboratory was used as an orientation session to familiarize the subjects with the study guidelines and testing procedures. This visit included an explanation of the treadmill tests and instructions for using MyFitnessPal for the recording of energy and macronutrient intake. Each subject also had the opportunity to walk on the treadmill at 4.8–6.4 km·h^−1^ and was asked to select a velocity within this range that they preferred to use during the 60-min exercise bouts of visits 2–4.

#### 2.2.2. Visit 2–4: Exercise Tests with Supplementation

##### Supplementation Protocol

Subjects were assigned to ingest (in a randomized and double-blind matter): (1) one serving of the preworkout supplement (Evlution Nutrition, Sunrise, FL, USA), (2) 6 mg caffeine anhydrous (Nutricost, Orem, UT, USA) per kg of body mass, (3) one serving of the placebo (noncaloric powdered drink mix matched with the preworkout supplement for flavor and consistency) (Walmart Stores, Inc., Bentonville, AR, USA) with 6 ounces of water 30 min prior to the exercise protocol. Ingredients of the preworkout supplement included: (1) a matrix (2016 mg) of betaine anhydrous, choline bitartrate, l-tyrosine, agmatine sulfate, alpha-glycerolphosphorylcholine, and huperzia serrata extract; (2) beta-alanine (1.6 g); (3) conjugated linoleic acid (500 mg); (4) l-carnitine l-tartrate (500 mg); (5) Coleus extract (as Coleus forskohli) (root) (100 mg); (6) Capsimax^®^ Cayenne (capsicum annuum) fruit extract (25 mg); (7) Yohimbe extract (as Pausinystalia yohimbe) (bark) (40 mg); (8) a blend (260 mg) of natural caffeine (from coffee bean) and green tea extract (standardized for EGCG) (leaf); (9) niacin (35 mg); (10) vitamin B6 (2 mg); (11) folic acid (800 mcg); and (12) vitamin B12 (25 mcg). The caffeine anhydrous (6 mg/kg of body mass) was mixed with one serving of the placebo to control for flavor and consistency.

##### Treadmill Exercise Tests

Subjects walked on a treadmill (Woodway Desmo HP, Waukesha, WI, USA) for 60 min at a predetermined self-selected velocity of 4.8–6.4 km·h^−1^. This predetermined velocity was held constant throughout the three 60-min exercise bouts. Prior to each test, the subjects were fitted with a heart rate monitor (Polar Electro Inc., Lake Success, NY, USA) and connected to a metabolic cart (TrueOne 2400, Parvo Medics, Sandy, UT, USA) with a 7450 Series Silicone V2 oro-nasal mask with headgear (Hans Rudolph, Inc., Shawnee, KS, USA) for the measurement of gas exchange. Blood pressure was measured with an inflatable sphygmomanometer (Medline Industries, Inc., Northfield, IL, USA) and stethoscope (Littmann, 3M Company, Maplewood, MN, USA) prior to ingesting the supplement condition (PRE) and 5-min postexercise (POST). Gas analyzers and flow volume of the metabolic cart were calibrated as previously described [24]. Oxygen uptake ($\dot{V}O$_2_), pulmonary ventilation ($\dot{V}$_E_), carbon dioxide output ($\dot{V}$CO_2_), respiratory exchange ratio (RER), and heart rate (HR) were recorded as 10-min averages throughout the test.

##### Estimation of Energy Expenditure and Substrate Utilization

Energy expenditure was estimated using the equations of Jeukendrup and Wallis [25]. Substrate utilization was estimated using the equations of Péronnet and Massicotte [26].

##### Likert Scale and Questionnaire

A five-point Likert scale questionnaire was used to determine feelings of fatigue, alertness, energy, and focus. The subjects circled the number that corresponded with their feelings for each category ranging from 1 (lowest feeling level) to 5 (highest feeling level).

##### Food Logs

Two-day food logs were recorded before each visit to the laboratory using MyFitnessPal (Baltimore, MD, USA) to assess total energy and macronutrient (carbohydrates, fat, and protein) intake average per day.

##### Statistical Analyses

Separate two-way analysis of variance (ANOVAs) with repeated measures were used to compare energy expenditure, $\dot{V}O$_2_, $\dot{V}$_E_, $\dot{V}$CO_2_, RER, fat and carbohydrate oxidation, and HR values among the conditions (caffeine, preworkout, and placebo) at the common time points (10, 20, 30, 40, 50, and 60 min) of the treadmill tests. When appropriate, follow-up tests included one-way ANOVAs with repeated measures and paired sample *t*-tests with Bonferroni corrections (0.05/3 = 0.0167). An alpha of *p* < 0.05 was considered statistically significant for all interaction effects and follow-up ANOVAs. Subjective feelings of fatigue, energy, focus, and alertness were assessed across supplement conditions and time with Friedman tests and follow-up Wilcoxon Signed Ranks test when appropriate. In addition, separate one-way ANOVAs with repeated measures were used to compare the total energy (kilocalories) and macronutrient (grams of protein, carbohydrate, and fat) intakes per day among the conditions.

## 3. Results

No treatment order effects (*p* > 0.05) were found across the three laboratory visits for all dependent variables.

### 3.1. Macronutrient Data

There were no significant differences among conditions for total energy intake (*F*(2.22) = 0.447; *p* = 0.646; partial η^2^ = 0.039), fat (*F*(2.22) = 0.422; *p* = 0.661; partial η^2^ = 0.037), carbohydrate (*F*(2.22) = 0.906; *p* = 0.419; partial η^2^ = 0.076), or protein (*F*(2.22) = 1.419; *p* = 0.263; partial η^2^ = 0.114) consumed each day (Table 1).

### 3.2. Metabolic Data

**Figure 2:**
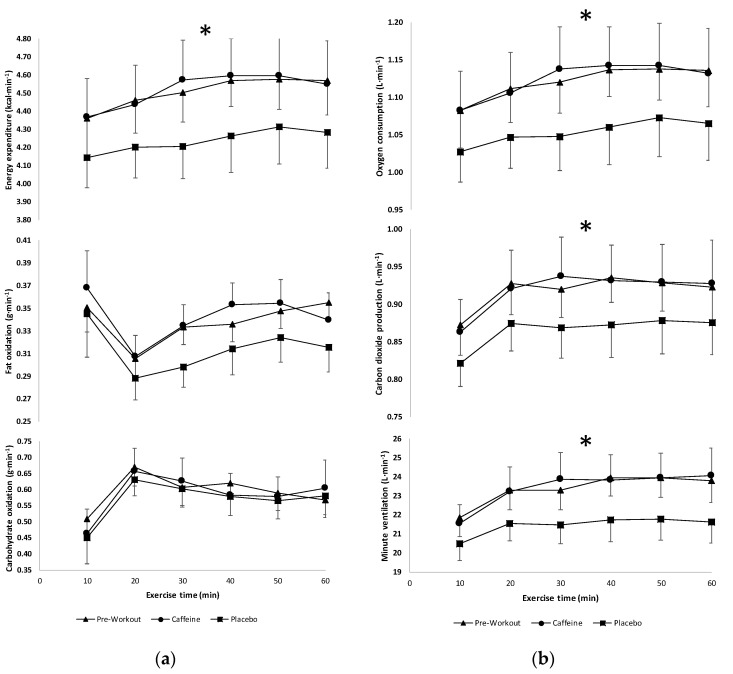
(**a**) Energy expenditure and substrate utilization values (mean ± SD) during 60 min of exercise among supplement conditions (n = 12). (**b**) Gas exchange and ventilatory values (mean ± SE) during 60 min of exercise among supplement conditions (n = 12). * Significant main effect for condition (Preworkout and Caffeine conditions > Placebo condition).

The results of the two-way repeated measures ANOVA indicated there was no significant interaction (*F*(10,110) = 0.397, *p* = 0.946, partial η^2^ = 0.035) for energy expenditure, but there were significant main effects for condition (*F*(2.22) = 9.322, *p* = 0.001, partial η^2^ = 0.459) and time (*F*(5.55) = 6.119, *p* < 0.001, partial η^2^ = 0.357). Follow-up paired-samples *t*-tests indicated that the preworkout and caffeine conditions resulted in significantly greater increases in energy expenditure (collapsed across time) compared to placebo (Figure 2). For rates of carbohydrate oxidation, there was no significant interaction (*F*(10,110) = 0.471, *p* = 0.906, partial η^2^ = 0.041) or main effects for condition (*F*(2.22) = 0.103, *p* = 0.903, partial η^2^ = 0.009), but there were main effects for time (*F*(5.55) = 6.535, *p* < 0.001, partial η^2^ = 0.373) (Figure 2). For rates of fat oxidation, there was no significant interaction (*F*(10,110) = 0.874, *p* = 0.560, partial η^2^ = 0.074) or main effects for condition (*F*(2.22) = 1.198, *p* = 0.321, partial η^2^ = 0.098), but there were main effects for time (*F*(5.55) = 4.910, *p* = 0.001, partial η^2^ = 0.309) (Figure 2). In addition, there was no significant interaction (*F*(10,110) = 0.336, *p* = 0.959, partial η^2^ = 0.032) for $\dot{V}O$_2_ among the different conditions at the common time points, but there were significant main effects for condition (*F*(2.22) = 8.918, *p* = 0.001, partial η^2^ = 0.448) and time (*F*(5.55) = 6.427, *p* < 0.001, partial η^2^ = 0.369). Follow-up paired-samples *t*-tests indicated that the preworkout and caffeine conditions resulted in significantly greater increases in $\dot{V}O$_2_ (collapsed across time) compared to placebo (Figure 2). For $\dot{V}$CO_2_, there was no significant interaction (*F*(10,110) = 0.242, *p* = 0.991, partial η^2^ = 0.022), but there were significant main effects for condition (*F*(2.22) = 4.069, *p* = 0.031, partial η^2^ = 0.270) and time (*F*(5.55) = 7.804, *p* < 0.001, partial η^2^ = 0.415). Follow-up paired-samples *t*-tests indicated that the preworkout and caffeine conditions resulted in significantly greater increases in $\dot{V}$CO_2_ (collapsed across time) compared to placebo (Figure 2). For $\dot{V}$_E_, there was no significant interaction (*F*(10,110) = 0.664, *p* = 0.773, partial η^2^ = 0.055), but there were significant main effects for condition (*F*(2.22) = 8.172, *p* = 0.002, partial η^2^ = 0.426) and time (*F*(5.55) = 5.923, *p* < 0.001, partial η^2^ = 0.350). Follow-up paired-samples *t*-tests indicated that the preworkout and caffeine conditions resulted in significantly greater increases in $\dot{V}$_E_ (collapsed across time) compared to placebo (Figure 2). For RER, there was no significant interaction (*F*(10,110) = 0.519, *p* = 0.874, partial η^2^ = 0.045) or main effect for condition (*F*(2.22) = 0.143, *p* = 0.868, partial η^2^ = 0.013), but there was a main effect for time (*F*(5,55) = 5.472, *p* < 0.001, partial η^2^ = 0.332).

### 3.3. Subjective Measures of Fatigue and Exertion

#### 3.3.1. Baseline

The results of the Friedman tests indicated that there were no differences among conditions for feelings of fatigue (χ^2^ = 0.87, *p* = 0.648), energy (χ^2^ = 2.14, *p* = 0.343), focus (χ^2^ = 0.67, *p* = 0.717), or alertness (χ^2^ = 1.30, *p* = 0.522) at baseline (Table 2).

#### 3.3.2. 30-Min Postingestion

At 30-min postingestion, there were significant differences among conditions for feelings of focus (χ^2^ = 10.42, *p* = 0.005) and alertness (χ^2^ = 6.65, *p* = 0.036), but not for fatigue (χ^2^ = 0.87, *p* = 0.648) or energy (χ^2^ = 4.39, *p* = 0.112). The follow-up Wilcoxon Signed Ranks tests indicated that the preworkout condition resulted in greater (*p* = 0.005) feelings of focus than the placebo condition, but there were no differences between the preworkout and caffeine conditions (*p* = 0.157), or caffeine and placebo conditions (*p* = 0.070). For feelings of alertness, the preworkout condition resulted in greater (*p* = 0.015) feelings of focus than the placebo condition, but there were no differences between the preworkout and caffeine conditions (*p* = 0.234), or caffeine and placebo conditions (*p* = 0.107) (Table 2).

#### 3.3.3. Exercise

There were significant differences among conditions for feelings of fatigue (χ^2^ = 6.64, *p* = 0.036), but not for energy (χ^2^ = 0.929, *p* = 0.629), focus (χ^2^ = 4.00, *p* = 0.135), or alertness (χ^2^ = 3.81, *p* = 0.149) at the 30-min time point of exercise (Table 2). The follow-up Wilcoxon Signed Ranks tests for feelings of fatigue indicated that the preworkout condition resulted in significantly (*p* = 0.014) lower feelings of fatigue than the placebo condition, whereas there was no difference (*p* = 0.557) between the caffeine and placebo conditions (Table 2).

### 3.4. Heart Rate and Blood Pressure

**Figure 3:**
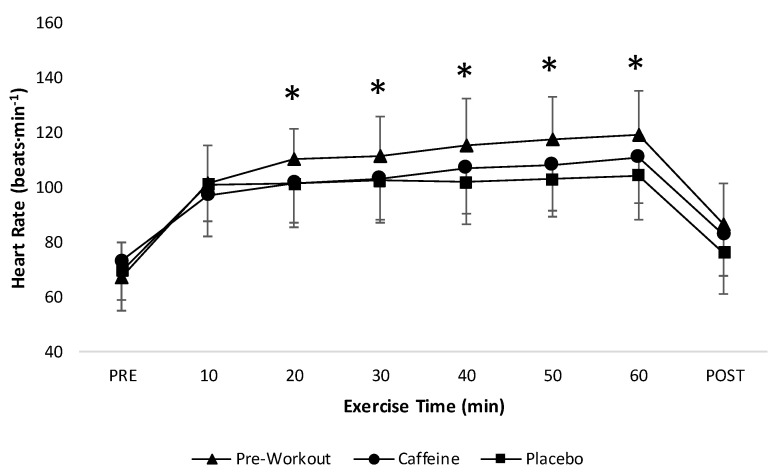
Heart rate values (mean ± SD) during 60 min of exercise among supplement conditions (*n* = 12). * Preworkout significantly (*p* < 0.05) greater than Caffeine and Placebo conditions.

For heart rate, there was a significant interaction (*F*(14.77) = 4.523; *p* < 0.001; partial η^2^ = 0.291) for condition (preworkout, caffeine, and placebo) across the common time points (baseline; 10, 20, 30, 40, 50, and 60-min; and postexercise). Follow-up one-way repeated measures ANOVAs indicated there were significant differences among condition at the 20 (*p* = 0.020, partial η^2^ = 0.299), 30 (*p* = 0.027, partial η^2^ = 0.281), 40 (*p* = 0.005, partial η^2^ = 0.377), 50 (*p* < 0.001, partial η^2^ = 0.114), and 60-min (*p* = 0.002, partial η^2^ = 0.440) time points, but not at baseline (*p* = 0.265, partial η^2^ = 0.114), 10-min (*p* = 0.085, partial η^2^ = 0.201), and postexercise (*p* = 0.086, partial η^2^ = 0.200) (Figure 3). For systolic blood pressure, there was no significant condition x time interaction (*F*(2.22) = 0.779; *p* = 0.471; partial η^2^ = 0.066) or main effect for condition (*F*(2.22) = 0.103; *p* = 0.903; partial η^2^ = 0.009), but there was a main effect for time (*F*(1.11) = 10.480; *p* = 0.008; partial η^2^ = 0.488) between baseline (123 ± 3 mmHg) and postexercise (127 ± 5 mmHg) values (Table 3). The results of the two-way repeated-measures ANOVA for diastolic blood pressure also indicated there was no significant condition x time interaction (*F*(2.22) = 0.244; *p* = 0.785; partial η^2^ = 0.022) or main effect for condition (*F*(2.22) = 1.892; *p* = 0.174; partial η^2^ = 0.147), but there was a main effect for time (*F*(1.11) = 6.828; *p* = 0.024; partial η^2^ = 0.383) between baseline (75 ± 4 mmHg) and postexercise (77 ± 4 mmHg) values (Table 3).

## 4. Discussion

The main finding of the present study indicated that the preworkout supplement significantly increased energy expenditure to a similar extent as 6 mg·kg^-1^ of caffeine during 60 min of low-intensity exercise. These results were surprising given that the caffeine content (<260 mg or <3.1 mg·kg^−1^) of the preworkout supplement was approximately half of the caffeine condition (496 ± 52 mg or 6.0 mg·kg^−1^). Previous studies [23,27,28,29] have shown that preworkout supplements provide positive effects on resting energy expenditure across a wide range of caffeine content (150–340 mg), whereas preworkout supplements with these same doses (i.e., 150 and 300 mg caffeine) had no effect during moderate-intensity exercise [24]. The influence of caffeine supplements alone on energy expenditure, however, appears to be dependent on the size of the relative dose. For example, 3 mg·kg^-1^ of caffeine has been shown [7,30] to have no effect compared to the 6 mg·kg^-1^ of caffeine used in the present study and other investigations [7] that demonstrated increases in energy expenditure. Collectively, these findings [7,23,24,27,28,29] suggested that other ingredients in the present preworkout supplement with moderate levels of caffeine (<260 mg or <3.1 mg·kg^−1^) may have provided a synergistic effect that increased energy expenditure.

Green tea comprises polyphenolic flavonoids known as catechins [31] that have been shown to enhance catecholamine activity through the inhibition of catechol-o-methyl transferase (COMT) [32]. Specifically, green tea extract has been demonstrated to increase total energy expenditure [33], total fat oxidation [31,34], and reduce total 24-h respiratory quotient [33]. Although green tea extract was included in a proprietary blend with caffeine at unknown quantities in the present preworkout supplement, these findings [31,32,33,34] suggest a possibility for increasing metabolic rate. Another ingredient in the preworkout was Capsicum annuum (25 mg) that contains capsainoids which activate sympathetically-mediated brown adipose tissue thermogenesis, thereby increasing whole-body energy expenditure [35]. Previous studies have shown that a single meal supplemented with capsicinoids resulted in increased energy expenditure and lipid oxidation [36,37]. Although there are limited data on the effects of Yohimbe extract as a nutritional supplement, it is an alpha-2-adrenrenoceptor blocking agent [38] that causes central nervous system stimulation [39]. Thus, due to the relatively low amount of caffeine content (<260 mg or <3.1 mg·kg^−1^) in the preworkout supplement used in the current study, which previously has not been shown to influence metabolism in the present preworkout supplement, it is likely that the inclusion of these ingredients (Green tea extract, Capsicum annuum, and Yohimbe extract) provided a synergistic effect for increasing energy expenditure to the same extent as the caffeine condition.

In the present investigation, the preworkout and caffeine supplements also increased $\dot{V}O$_2_, $\dot{V}$CO_2_, and $\dot{V}$_E_, but had no effect on RER and rates of carbohydrate and fat oxidation compared to placebo. While there is limited research on the effects of preworkout supplementation on aerobic exercise, the results of the present study are inconsistent with those previously observed in regard to oxygen consumption. For example, Erickson and colleagues [24] reported no significant changes in $\dot{V}O$_2_ during 30-min treadmill running bouts at 90% of ventilatory threshold following the ingestion of one and two doses of a preworkout supplement containing caffeine (150 and 300 mg, respectively). In addition, the administration of 6 mg·kg^−1^ of caffeine alone has been shown to increase $\dot{V}O$_2_ during treadmill walking in females [7], whereas 3 mg·kg^−1^ of caffeine has resulted in no change [7]. Thus, our findings of increased $\dot{V}O$_2_ in the preworkout and caffeine conditions further support a possible synergistic effect from the additional substances (i.e., green tea extract, Capsicum annuum, and Yohimbe extract) included in the current multi-ingredient preworkout supplement.

Despite the increases in energy expenditure and $\dot{V}O$_2_, however, we observed no significant differences among conditions (preworkout, caffeine, placebo) for RER or carbohydrate and fat oxidation. Our findings for lack of change in RER under the preworkout and caffeine conditions can likely be explained by concurrent increases in $\dot{V}O$_2_ and $\dot{V}$CO_2_. Erickson and colleagues [24] also reported no effect on substrate utilization following the ingestion of a preworkout supplement in trained females during treadmill running. Similarly, Jung and colleagues [28] observed no change in RER in recreationally active males and females during resting metabolic rate tests following the ingestion of preworkout with *p*-synephrine. In contrast, caffeine has been shown to increase intramuscular fat oxidation during leg ergometer cycling at a dose of 5 mg·kg^−1^ [6]. Hulston and Jeukendrup [40], however, reported that caffeine at a dose of 5.3 mg·kg^−1^ ingested along with a glucose solution had no effect on total body-fat oxidation during endurance exercise, subsequently affirming our findings. The results of the present investigation align with previous research [6,24,28,40] in regard to substrate utilization and RER following preworkout and caffeine supplementation, likely due to concurrent increases in $\dot{V}O$_2_ and $\dot{V}$CO_2_.

When comparing subjective feelings of fatigue, focus, alertness, and energy, there were no differences among baseline measures. However, thirty minutes postingestion (immediately preexercise), alertness and focus were significantly higher in the preworkout supplement compared to the placebo. With examination of the self-reported psychological variables across multiple time points, the multi-ingredient preworkout supplement condition caused a significant reduction in feelings of fatigue during exercise when compared to the placebo, while caffeine saw no significant difference. Preworkout supplementation has been shown to influence subjective feelings such as focus and energy, without reported changes in subjective feelings of fatigue or alertness [41]. In addition, Walsh and colleagues [17] examined the effects of a multi-ingredient preworkout supplement on treadmill run time to exhaustion, reporting an increased time to exhaustion by 12.5% in the supplement condition. The authors of [17] also observed a significant reduction in fatigue pre-exercise compared to the placebo, attributing it to the combination of multiple ingredients within the supplement. While caffeine has been known to increase feelings of focus and alertness [42], and reduce feelings of exertion [11,12] when ingested in isolation, the inclusion of other ingredients combined with caffeine likely resulted in the reduced feelings of fatigue during exercise.

In the present investigation, the preworkout condition led to significantly greater increases in HR during the 60 min of treadmill exercise compared to the caffeine and placebo conditions (Figure 3). Given that the amount of caffeine was approximately twice as high in the caffeine condition (496 ± 52 mg or 6.0 mg·kg^−1^) than the preworkout condition (<260 mg or <3.1 mg·kg^−1^), it is unlikely to be responsible for this effect on HR. Consistent with our findings, the administration of caffeine at 6.0 mg·kg^−1^ has been shown to have no influence on HR at rest or during submaximal exercise [43]. In contrast to our findings for the present preworkout supplement, however, Bergstrom et al. [44], and Erickson et al. [24], reported no change in resting or submaximal HR with the supplementation of caffeine-based preworkouts. Collectively, the current findings and those of others [24,43,44] suggested that the increase in HR observed in the preworkout condition was attributable to an ingredient(s) other than caffeine. It is also important to note that the stimulatory effect on HR in the preworkout condition did not translate into changes in systolic or diastolic blood pressure from pre- to postexercise (Table 3). Given these inconsistent findings and the difficulty in identifying the substances responsible, individuals hoping to avoid any type of cardiovascular effect from preworkout supplementation should practice caution with the selection and administration of their product.

The present study had several limitations. Specifically, the preworkout supplement had caffeine included in a proprietary blend with green tea extract. Thus, the exact amounts of both of these ingredients are unknown, making comparisons to other products difficult. In addition, based on an average half-life of 5 h for caffeine [3], the average dose of caffeine in this study (mean ± SD = 496 ± 52 mg) following a 48–72 h washout period would result in approximately <1 mg and <0.04 mg remaining in the system, respectively. Furthermore, it is unclear if the effects of the present preworkout supplement during low-intensity exercise can be extended to moderate and high levels of intensity. Lastly, it is uncertain if the current findings reported for male participants could be expected in females due to potential gender differences.

## 5. Conclusions

In summary, acute ingestion of the multi-ingredient preworkout supplement that contained moderate levels of caffeine (<260 mg or <3.1 mg·kg^−1^) significantly increased (collapsed across conditions) energy expenditure, $\dot{V}O$_2_, $\dot{V}$CO_2_, and $\dot{V}$_E_ to the same extent as 6 mg·kg^−1^ of caffeine (496 ± 52 mg). Neither the preworkout or caffeine conditions, however, had any effect on rates of fat or carbohydrate oxidation or RER. In addition, the preworkout supplement increased feelings of alertness and focus 30-min postingestion and decreased feelings of fatigue during exercise, whereas there were no changes for the caffeine condition compared to placebo. These findings indicated that the present preworkout supplement contains ingredients other than caffeine that provided a synergistic effect for increasing metabolic rate and measures of gas exchange while improving feelings of alertness, focus, and fatigue. Future studies should examine the individual effects of these ingredients (i.e., green tea extract, Capsicum, and Yohimbe extract) on metabolic and psychological variables of exercise performance. For the general consumer or practitioner, our findings demonstrated that the current preworkout supplement may be more favorable than caffeine for improving feelings of alertness, focus, and fatigue while maintaining a similar elevated metabolic rate during exercise.

## Figures and Tables

**Table 1:** Two-day average (mean ± SD) for total calories and macronutrients consumed during the preworkout, caffeine, and placebo conditions (n = 12).

	Preworkout	Caffeine	Placebo
Total calories (kcals·d^−1^)	2086 ± 876	2172 ± 792	2101 ± 895
Carbohydrates (g·d^−1^)	209 ± 105	226 ± 82	211 ± 96
Fat (g·d^−1^)	87 ± 38	93 ± 34	87 ± 37
Protein (g·d^−1^)	97 ± 51	93 ± 34	87 ± 37

Note: There were no significant (*p* > 0.05) differences among conditions for kcals or macronutrients.

**Table 2:** Likert scale values (mean ± SD) for the psychological variables among supplement conditions at the 30-min time point of exercise (n = 12).

	Preworkout	Caffeine	Placebo
Baseline			
Energy	2.8 ± 0.7	2.8 ± 0.9	2.6 ± 0.9
Fatigue	2.2 ± 0.9	2.3 ± 0.8	2.0 ± 0.7
Alertness	3.1 ± 0.7	2.8 ± 0.8	2.8 ± 1.0
Focus	3.3 ± 0.6	3.2 ± 0.8	3.0 ± 1.0
30-Min Postingestion			
Energy	3.6 ± 0.7	3.3 ± 0.7	3.0 ± 0.7
Fatigue	2.2 ± 0.9	2.0 ± 0.7	2.0 ± 0.7
Alertness	4.0 ± 0.7 *	3.6 ± 0.9	3.5 ± 1.2
Focus	4.2 ± 0.7 *	3.8 ± 0.9	3.3 ± 0.6
Exercise			
Energy	3.8 ± 0.6	3.7 ± 0.8	3.5 ± 0.8
Fatigue	2.0 ± 0.9 *	2.3 ± 1.0	2.5 ± 0.8
Alertness	4.0 ± 0.7	3.6 ± 0.9	3.5 ± 1.2
Focus	4.3 ± 0.8	3.8 ± 0.9	3.8 ± 1.2

* Significantly (*p* < 0.05) different than placebo conditions.

**Table 3:** Blood pressure responses (mean ± SD) among the supplement conditions (n = 12).

Variable	Preworkout	Caffeine	Placebo
Systolic baseline (mmHg)	122.3 ± 4.3	123.2 ± 4.1	123.5 ± 3.5
Systolic postexercise (mmHg)	128.3 ± 11.0	126.2 ± 4.6	126.3 ± 4.4
Diastolic baseline (mmHg)	73.2 ± 4.9	73.3 ± 6.6	75.0 ± 4.6
Diastolic postexercise (mmHg)	75.5 ± 4.9	78.7 ± 6.8	76.3 ± 4.2

Note: There were no significant (*p* > 0.05) interactions or main effects for condition or time for both systolic and diastolic blood pressure responses.
